# Radionuclide imaging of apoptosis for clinical application

**DOI:** 10.1007/s00259-021-05641-4

**Published:** 2021-12-07

**Authors:** Xiyi Qin, Han Jiang, Yu Liu, Hong Zhang, Mei Tian

**Affiliations:** 1 Department of Nuclear Medicine and PET Center, The Second Affiliated Hospital of Zhejiang University School of Medicine, 88 Jiefang Road, Hangzhou, 310009 Zhejiang China; 2 Institute of Nuclear Medicine and Molecular Imaging of Zhejiang University, Hangzhou, China; 3 Key Laboratory of Medical Molecular Imaging of Zhejiang Province, Hangzhou, China; 4 PET-CT Center, Fujian Medical University Union Hospital, Fuzhou, 350001 China; 5 College of Biomedical Engineering & Instrument Science, Zhejiang University, Hangzhou, China; 6 Key Laboratory for Biomedical Engineering of Ministry of Education, Zhejiang University, Hangzhou, China

**Keywords:** Apoptosis, Single-photon emission computed tomography (SPECT), Positron emission tomography (PET), Radionuclide imaging

## Abstract

Apoptosis was a natural, non-inflammatory, energy-dependent form of programmed cell death (PCD) that can be discovered in a variety of physiological and pathological processes. Based on its characteristic biochemical changes, a great number of apoptosis probes for single-photon emission computed tomography (SPECT) and positron emission tomography (PET) have been developed. Radionuclide imaging with these tracers were potential for the repetitive and selective detection of apoptotic cell death in vivo, without the need for invasive biopsy. In this review, we overviewed molecular mechanism and specific biochemical changes in apoptotic cells and summarized the existing tracers that have been used in clinical trials as well as their potentialities and limitations. Particularly, we highlighted the clinic applications of apoptosis imaging as diagnostic markers, early-response indicators, and prognostic predictors in multiple disease fields.

## Introduction

Several billions of normal cells died daily in the human body without being recognized by the immune system, which was of great importance in the whole-body homeostasis preservation [1]. Apoptosis, a natural, non-inflammatory, energy-dependent form of programmed cell death (PCD), was considered to play an indispensable role in it. First described by Kerr and colleagues in 1972 [2], apoptosis was known to participate in varieties of normal physiological processes, such as embryonic development and immune system regulation [3]. Besides, the close association between apoptosis dysregulation and certain disease was also indicted. Excessive apoptosis, for example, was involved in a series of neurodegenerative diseases, while tumor cells tended to evade apoptosis for promoting tumor progression and drug resistance [3, 4]. Therefore, it was believed that testing and monitoring apoptosis levels in these patients can not only provide very useful information on the disease diagnosis and staging but also conduce to the efficacy assessment of their treatments [5]. Although histopathological methods, such as terminal deoxynucleotidyl transferase (TdT)–mediated dUTP-biotin nick end labeling (TUNEL) assays, have long been served as the “gold standard” for qualitative and quantitative analysis of tissue apoptosis status, their clinical availability was still very limited [4]. It was the invasive damage of histopathological biopsies as well as the need for persistent concerns on the lesion apoptosis level that significantly challenged their developments and more extensive applications in clinic [4, 6]. Thus, ongoing efforts were required to promote clinical pathology toward the pattern of cross-scale, multi-mode “transparent pathology,” allowing for noninvasive and repeatable in vivo detection of various biological features of lesions, including apoptosis levels [7].

Apoptosis imaging was such a noninvasive procedure that enabled in vivo exploration of apoptosis level during disease occurrence, development, or remission [5]. Among various apoptosis visualization techniques, radionuclide imaging, such as single photon emission computed tomography (SPECT) and positron emission tomography (PET), showed the most advantage in precise detection of apoptosis at the early stage. Using molecular-targeted radiopharmaceuticals, we were able to finely diagnose apoptosis-related disease, predict damage severity, and assess therapy response in real time [5]. Taking the antitumor efficacy evaluation as an example, tumor cells were known to undergo apoptosis within 24 h after treatment instigation. However, it has been confirmed that traditional criteria, such as computed tomography (CT) and magnetic resonance imaging (MRI), required at least 2–3 months to identify apparent anatomical alterations [5, 8]. ^18^F-fluorodeoxy-glucose (^18^F-FDG) PET, by comparison, has long been employed as a sensitive method for early curative effect monitoring. The limitations of ^18^F-FDG were also apparent, mainly due to nonspecific accumulation and inability to detect low metabolic lesion [5, 8]. A promising solution to this dilemma was to use SPECT/PET probes specifically targeting apoptosis, which were of great clinical value in avoiding the unnecessary side effects caused by ineffective treatment for several weeks [3].

Over the past few decades, emerging numbers of radiopharmaceuticals targeting apoptosis have been developed. However, up to now, the utility of these probes was still largely confined to preclinical research studies. This was partly due to their objective limitations: a successful clinical apoptosis imaging agent should be an integration of various features, such as easy synthesis, sensitive and selective detection of the apoptotic cells, favorable biodistribution, and safety profiles in human bodies. Thus, more attention should be paid on the clinical practice of existing tracers. In-depth analysis as well as cross comparison of their strengths and shortcomings would be helpful to identify key issues as well as opportunities for improvement. In this review, we mainly summarized molecular mechanism and specific biochemical changes in apoptotic cells, provided an overview of several representative tracers which have been widely used in clinical trials to monitor these alterations, and discussed the potentialities and limitations of these existing probes, with a special emphasis on their roles as diagnostic markers, early-response indicators, and prognostic predictors in multiple medical fields.

## Process of apoptosis

**Fig. 1:**
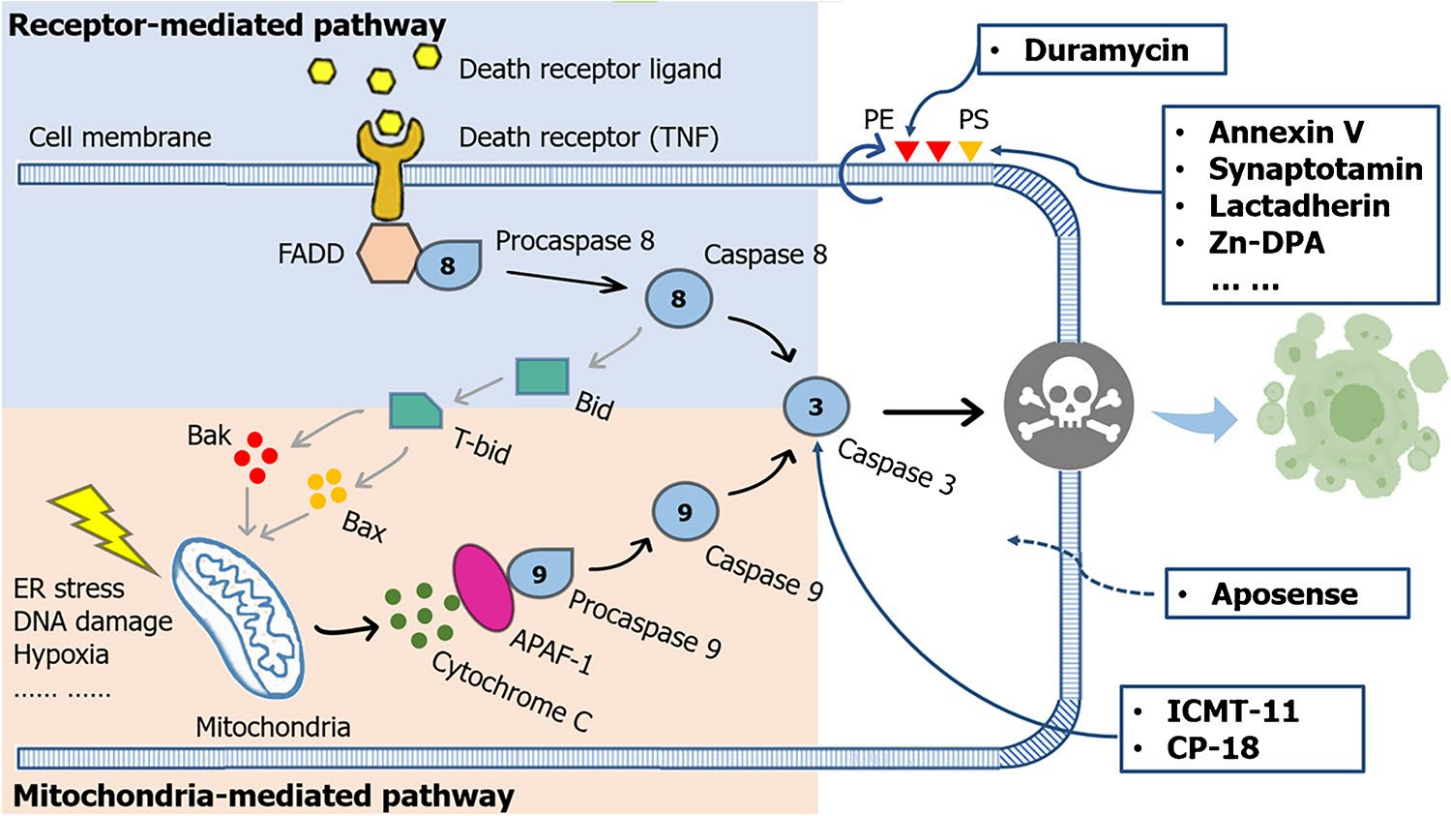
Pathways and imaging targets of apoptosis. The extrinsic pathway and intrinsic pathway converge at the execution phase, where caspase-3 is activated so as to finally result in apoptosis. The extruded phospholipid (i.e., PS and PE), activated caspase 3, and altered membrane permeability can be targeted by various radiotracers for noninvasive imaging of apoptosis

There were two major pathways for apoptosis initiation: the intrinsic and extrinsic pathways (Fig. 1). Mitochondrial or intrinsic pathway, induced by internal cellular stress signals such as endoplasmic reticulum (ER) stress, DNA damage, and hypoxia, was initiated with the elevated mitochondrial outer membrane permeabilization (MOMP) and subsequent release of mitochondrial cytochrome c. In cytoplasm, cytochrome c was quickly attached to the adaptor protein APAF-1, and then a complex called apoptosome was developed, through which the initiator caspase 9 was cleaved. Caspase 9 was a cysteine aspartate specific protease, which can trigger the executioner pathway once activated and finally induce apoptosis. The whole process is under the modulation of the Bcl-2 proteins family [3, 8, 9].

In extrinsic pathway, alternatively, it was the external cellular stimuli that finally triggered apoptosis process. Death receptors on cell surface became activated upon binding to death ligands, followed by the recruitment of adaptor molecule Fas-associated death domain (FADD) and pro-cysteine dependent aspartate-directed enzyme 8 (pro-caspase 8). As a result, a death-inducing signaling complex (DISC) on the cytosolic domain of the receptor can be found, and the caspase 8 was cleaved, leading to the sequential conversion or activation of the executioner pathway caspases. Interestingly, under certain conditions, caspase 8 was also able to induce the cytochrome c release and initiate mitochondrial-mediated pathway during extrinsic apoptosis; a crosstalk occurred when proapoptotic protein truncated-Bid (t-Bid) was cleaved by caspase 8 [3, 8, 9].

Moreover, as mentioned above, the intrinsic and extrinsic pathways converged at the execution phase, where caspase-3 was activated. Undergoing a series of morphological and biochemical alterations, such as the externalization of phosphatidylserine (PS) and phosphatidylethanolamine (PE) as well as acidification of the cell membrane, apoptosis cells were thought to disassemble from within via activated caspase 3, characterized by chromatin condensation, DNA/protein breakdown, cell shrinkage, and apoptotic bodies generation. Finally, dying cells externalizing PS as an “eat-me” signal were ingested and removed by macrophages without causing inflammation [3, 8, 9].

## Apoptosis imaging

Radionuclide imaging of apoptosis was a noninvasive test which can finely visualize, characterize, and measure the apoptosis level of lesions in vivo. The radiolabeling was commonly used to identify specific biochemical changes that occurred in apoptotic cells, thereby providing physiological information at the molecular level [10]. After administration of these radiotracers, apoptosis cells in vivo can be radiolabeled and then detected by radionuclide imaging. To date, a great number of molecular markers have been investigated for apoptosis imaging, including extruded PS/PE at the outer leaflet of plasma membrane, activated caspases in the intracellular compartment, apoptotic membrane imprint, and dissipated mitochondrial membrane potential [8]. Some of them have already been investigated in a series of clinical researches (Table 1).

**Table.1:** Molecular imaging of apoptosis in multiple disease fields

Medical field	Clinical application	Radiotracer	Reference
Healthy volunteers	Biodistribution, dosimetry, and safety	^99m^Tc-Annexin V, ^18^F-ML-10, ^18^F-ICMT-11, ^18^F-CP18	[18–20, 26, 29, 32]
Oncology	Identification of apoptosis in malignant tumors	^99m^Tc-Annexin V, ^18^F-ML-10	[38, 41, 42]
	Early assessment of response to therapy	^99m^Tc-Annexin V, ^18^F-ML-10, ^18^F-ICMT-11	[47–51, 89]
	Prediction of overall or progression-free survival	^99m^Tc-Annexin V	[55, 56]
Cardiology	Identification of apoptosis in heart failure	^99m^Tc-Annexin V	[67]
	Diagnosis and assessment of ARVC/D	^99m^Tc-Annexin V	[79]
	Location and assessment of cardiac infraction/reperfusion damage	^99m^Tc-Annexin V	[65, 66]
	Identification of apoptosis in unstable atherosclerotic plaque	^99m^Tc-Annexin V	[78]
	Diagnosis and assessment of cardiac allograft rejection	^99m^Tc-Annexin V	[90]
Neurology	Location and assessment of acute stroke	^99m^Tc-Annexin V	[69, 70]
	Differential diagnosis of AD and non-AD dementia	^99m^Tc-Annexin V	[83]
Others	Early assessment of response to therapy in Crohn’s disease	^99m^Tc-Annexin V	[46]
	Differential diagnosis of loosening and infection of prostheses	^99m^Tc-Annexin V	[85]

### Apoptosis imaging in healthy volunteers

Prior to abundant clinical trials, scientists quantified the biodistribution, dosimetry, and safety of relevant probes in human subjects, thus determining whether it was suitable for apoptosis imaging in patients.

#### Apoptosis imaging in healthy volunteers: targeting membrane asymmetry

Eukaryotic cell membrane was a highly specialized bilayer of asymmetrically distributed phospholipids [9]. The layout was not fixed as multiple cellular activities were accompanied with remarkable changes in cell membrane morphology or composition [9]. One of the defining alterations of apoptosis, for example, was the translocation and externalization of anionic phospholipids, including PE and PS [3]. Appearing early at the apoptosis cascade, this disturbed asymmetry can be served as a potential biomarker for radionuclide imaging of apoptosis due to the broad expression and easy accessibility of PE/PS [5]. Nowadays, a rich assortment of imaging probes targeting the externalized anionic phospholipids, such as Annexin V [11], synaptotagmin I derivatives [12], lactadherin [13], PS-binding peptides [14] and monoclonal antibody fragments [15], Zn-DPA complexes [16], as well as duramycin [17], have been developed for distinguishing of apoptotic cells.

**Fig. 2:**
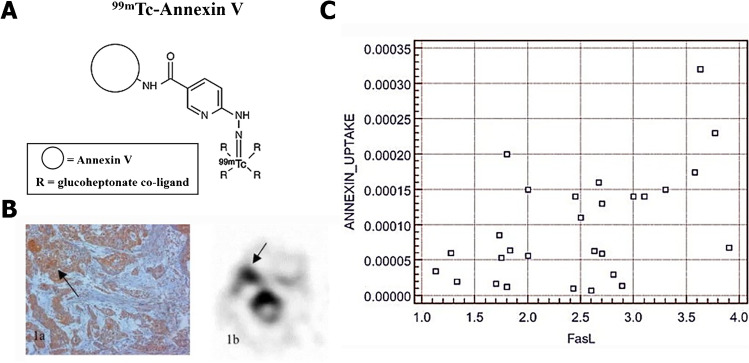
**A** Schematic diagram of chemical structure of ^99m^Tc-HYNIC-Annexin V. **B** Tumor with high ^99m^Tc-HYNIC-Annexin V uptake shows high FasL staining, and their linear relationship was shown in **C**. (The researches were originally published in Eur J Nucl Med Mol Imaging. 2004 Jul;31(7):1016–21 [39]. Reprint permission was obtained)

Out of these imaging agents, ^99m^Tc-Annexin V was by far the only one that has been extensively investigated and proceeded to the clinical stage of testing. Multiple types of chelators and co-ligands have been used by different groups for developing diverse ^99m^Tc-Annexin V radiopharmaceuticals. Each radioligand was accompanied by a distinct biological behavior, such as ^99m^Tc-BTAP-Annexin V, ^99m^Tc-i-Annexin V, and ^99m^Tc-HYNIC-Annexin V (Table 2). All of the three probes were favorable candidates for apoptosis imaging, with acceptable effect doses at 7.6 ± 0.5, 9.7 ± 1.0, and 11.0 ± 0.8 µSv/MBq, respectively [18–20]. However, their limitations were also evident. ^99m^Tc-BTAP-Annexin V was reported to hold the most complex and time-consuming synthesis process among three probes (about 75 min with relatively low radiochemical yields of 25 ~ 30%) [21], which greatly limited its clinical availability. While ^99m^Tc-i-Annexin V was expected to address some of the limitations of ^99m^Tc-BTAP-Annexin V, whose preparation was relatively fast and easy [19], new problems continued to crop up. Both ^99m^Tc-BTAP-Annexin V and ^99m^Tc-i-Annexin V were mainly excreted via urine and feces. Given their fast appearance in the intestines and extensive bowel excretion, it was difficult for using ^99m^Tc-BTAP-Annexin V and ^99m^Tc-i-Annexin V to visualize the abdominal region. Moreover, ^99m^Tc-i-Annexin V had longer half-time than ^99m^Tc-BTAP-Annexin V, which may cause higher radiation doses in its main accumulation organs, such as the bladder and the bowel. By comparison, ^99m^Tc-HYNIC-Annexin V (Fig. 2A) was excreted almost through the urine, which may indicate that it was a candidate more feasible for translation. However, as a large molecule (36 kDa) with positive charges, ^99m^Tc-HYNIC-Annexin V bound to PS in a calcium-dependent manner and inflicted a low lesion-to-background ratio owing to low tissue penetration [9]. Besides, all of the three probes showed significant nontarget organ radiation burden, especially at the kidneys and liver, making them not appropriate for serial imaging [6, 19]. Notably, since PS was also exposed on necrotic cells, ^99m^Tc-HYNIC-Annexin V may not be capable of discriminating between apoptosis and necrosis [3]. Therefore, a more advanced imaging approach with high sensitivity and specificity but reduced radiation burden was needed.

**Table.2:** Biodistribution and dosimetry of diverse ^99m^Tc-Annexin-V in humans

Radiopharmaceuticals		^99m^Tc-BTAP-Annexin V	^99m^Tc-i-Annexin V	^99m^Tc-HYNIC-Annexin V
Effective dose (µSv/MBq)		7.6 ± 0.5	9.7 ± 1.0	11.0 ± 0.8
Distribution (Gy/MBq)	Kidneys	63 ± 22	93 ± 24	196 ± 31
	Spleen	15 ± 3	22 ± 6	41 ± 12
	Liver	13 ± 3	17 ± 2	16.9 ± 1.3
Half-time (h)		16 ± 7	62 ± 13	69 ± 7
Excretion		Urine (73%)	Urine (75%)	Urine
		Feces (27%)	Feces (25%)	
Reference		[18]	[19]	[20]

Nowadays, several ^99m^Tc-Annexin V mutants have been developed and investigated in some animal models, which may represent a feasible optimization scheme [22–24]. ^99m^Tc-Annexin V-128, for instance, was demonstrated to possess the same or even higher accumulation in apoptosis tissues than ^99m^Tc-HYNIC-Annexin V, but with an 88% less renal retention of radioactivity [25]. In addition, the emergence of radiolabeled PS-targeting peptides provided another innovative direction for radionuclide imaging of apoptosis. These probes were expected to have simpler synthesis strategies, improved tissue penetration, faster blood clearance profile, and so on. Recently, scientists have also considered duramycin, a PE-binding tracer, as a promising agent for apoptosis imaging. It had a much lower molecular weight (2 kDa) than Annexin V (36 kDa), with an optimized pharmacokinetic pattern and higher target/background ratio. Unfortunately, none of these radiopharmaceuticals have been investigated in humans yet.

#### Apoptosis imaging in healthy volunteers: targeting cell membrane acidification

Previous studies have shown that the energy barrier of the cell membrane can be destroyed by the scrambling processes of early apoptosis (depolarization), accompanied with a significant reduction in the pH of the external membrane leaflet and cytoplasm (acidification) [9]. Although the mechanism of acidification and depolarization were not yet completely understood, Aposense family, a group of small amphipathic molecules, has been designed to identify the altered membrane permeability in apoptotic cells and then distinguish them from viable ones [5].

**Fig. 3:**
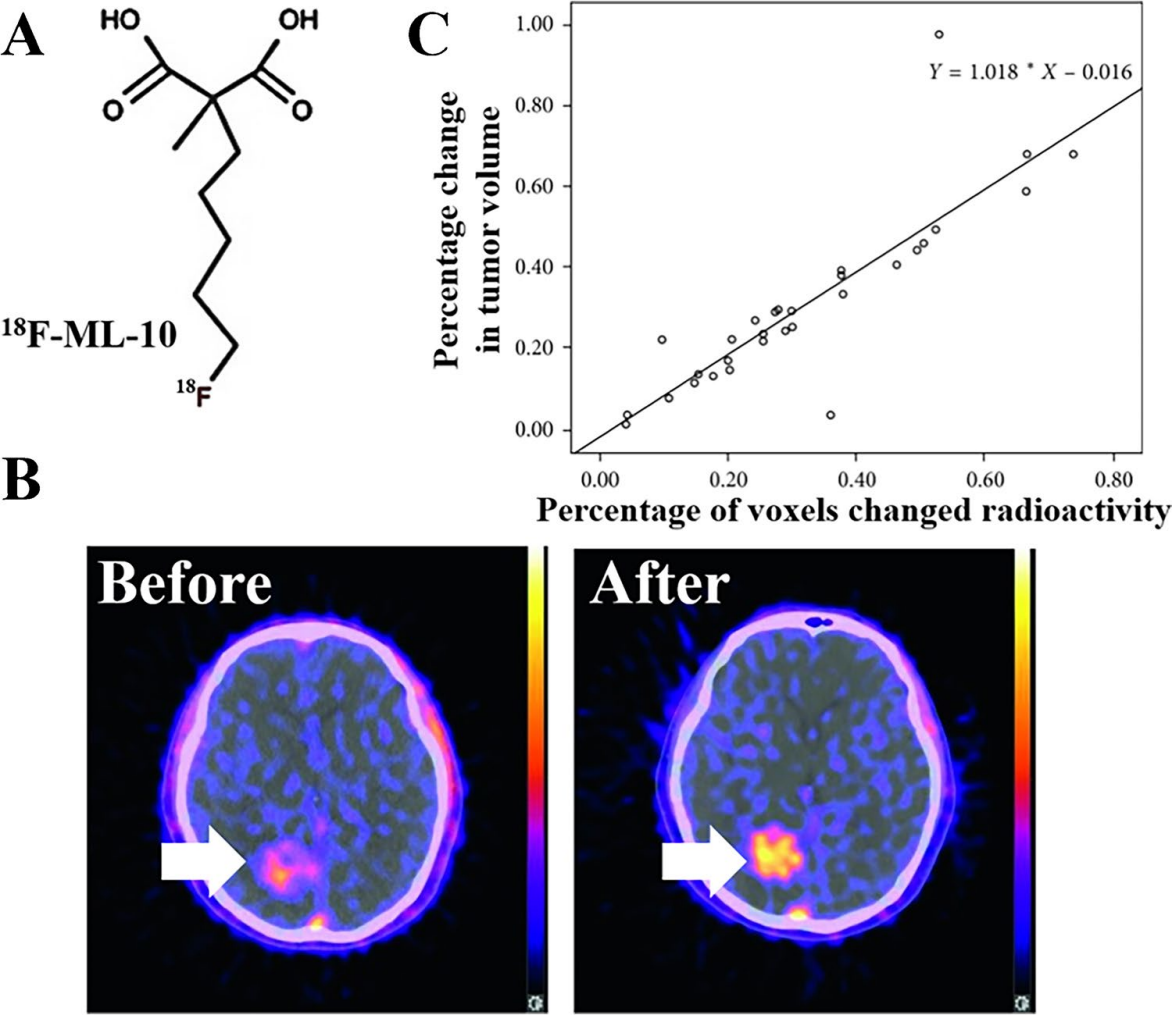
**A** Schematic diagram of chemical structure of ^18^F-ML-10. **B** Typical images of ^18^F-ML-10 PET/CT before (left) and after (right) CK treatment. **C** Correlation between early changes in ^18^F-ML-10 uptake and anatomic response in tumor volume. The Pearson correlation coefficient is *R* = 0.862, *p* < 0.05. (This research was originally published in Contrast Media Mol Imaging. 2018; 2018:9,365,174 [89])

Among these apoptosis tracers targeting apoptotic membrane imprint, ^18^F-ML-10 (Fig. 3A) was the only radiopharmaceutical that has been investigated in clinical research. It was highly stable in vivo followed by an average effective whole-body dose of 15.4 ± 3.7 μSv/MBq [26]. The clearance of this radiopharmaceutical was fast, with a half-life of 1.3 ± 0.1 h in the blood as well as 1.1 ± 0.2 h in other organs. Moreover, since the tracer was mainly excreted through the urine, it seemed that the urinary bladder was the dose-limiting organ that received the most radiation dose.

As opposed to ^99m^Tc-Annexin V, ^18^F-ML-10 born a higher sensitivity and faster blood clearance profile due to its smaller molecular size and minimal number of functional groups, which allowed for a more accurate quantitative evaluation of tracer uptake [8]. Moreover, ^18^F-ML-10 was confirmed to specifically detect apoptotic cells and discriminate them from other forms of cell death. Nevertheless, the concrete mechanism for this radioligand to bind with apoptotic cell was rather vague [4]. Some studies have also pointed out that the uptake of ^18^F-ML-10 is pH-sensitive [27]. That is to say: changes of the blood or extracellular pH may influence the nonspecific uptake of ^18^F-ML-10 in viable tissues. This may not only lead to the requirement of a high administered dose which may cause potential toxicity but also complicate the interpretation of ^18^F-ML-10 PET/CT images. Further study was needed to optimize the imaging characteristics of ^18^F-ML-10.

^18^F-ML-8 was an analog of ^18^F-ML-10, which had a lower molecular weight. It shared the same dynamic uptake process with ^18^F-ML-10 and both can differentiate apoptosis from necrosis. Moreover, according to a study performed by Hui Ma and colleagues [28], in a rat myocardial infarction model, ^18^F-ML-8 activity showed better correlation with apoptosis rates calculated from the TUNEL test than ^18^F-ML-10 uptake. Although requiring more evidences and clinical attempts, ^18^F-ML-8 may be potentially more suitable for clinical evaluation of apoptosis and deemed more feasible for translation.

#### Apoptosis imaging in healthy volunteers: targeting caspase

**Fig. 4:**
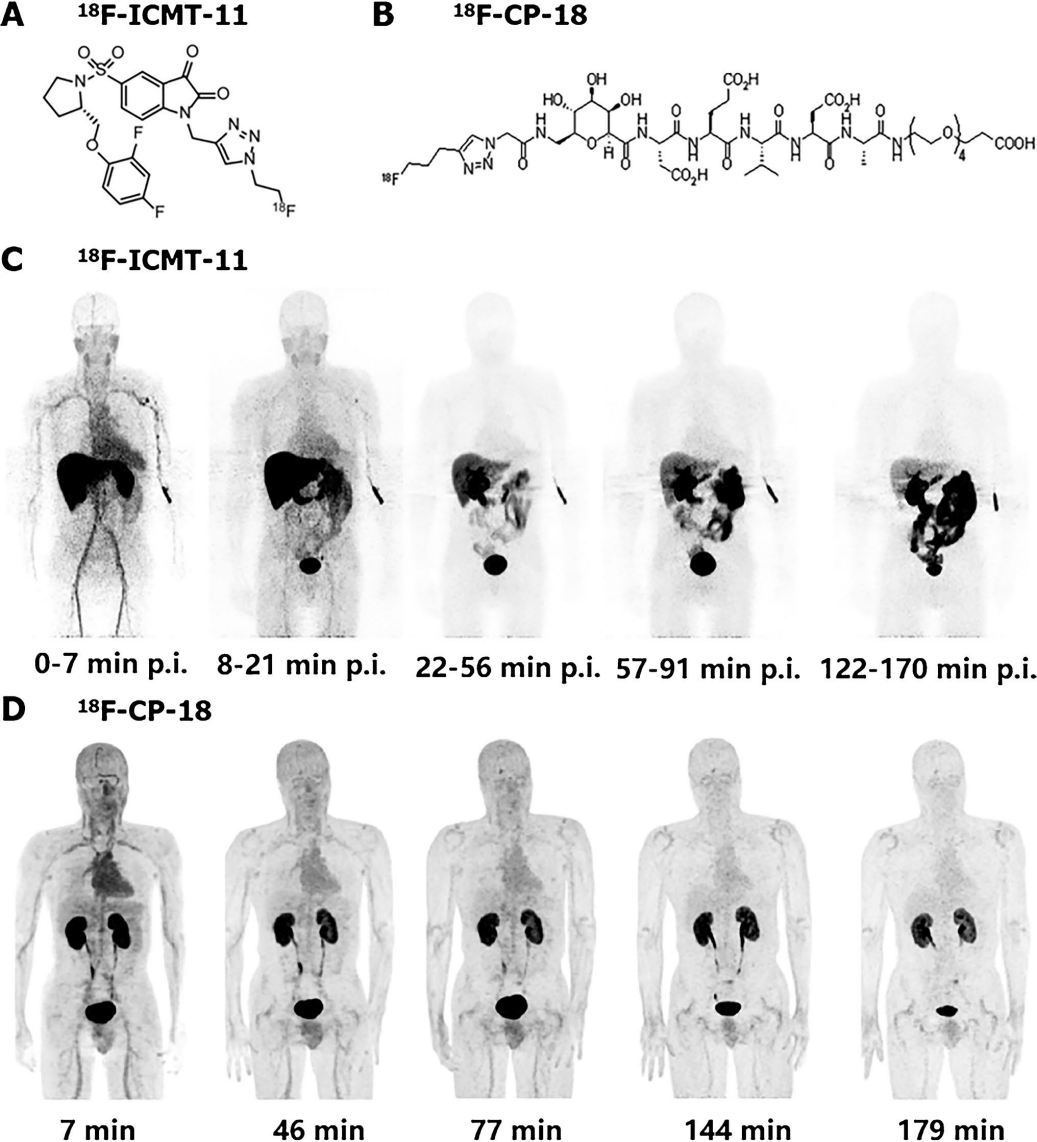
Chemical structure and biodistribution of two caspase targeting probes. Chemical structures of ^18^F-ICMT-11 (**A**) and ^18^F-CP-18 (**B**) are shown. There is rapid clearance of ^18^F-ICMT-11 in cranium and thorax approximately 20 min after injection and gallbladder bowel accumulations starts from about 30 min (**C**). In contrast, ^18^F-CP-18 shows a rapid clearance in all organs (**D**). (The researches were originally published in J Nucl Med. 2013 Sep;54(9):1551–6 [29] and J Nucl Med. 2013 Dec;54(12):2087–92 [32], respectively)

Caspase-3 was the main executer of apoptosis which may act as an ideal tool for apoptosis imaging [9]. Considering the different apoptosis initiation pathways ultimately converged at the execution phase, executive caspase-3 was capable of discerning apoptotic cells specifically even before morphological alterations [5, 8]. In vivo imaging of apoptosis through targeting activated caspases was possible via two different approaches: use of caspase inhibitors or substrates. To date, multiple types of radiolabeled caspase-3 ligands have been synthesized and some of them were already used in human bodies (Fig. 4).

Recently, ^18^F-ICMT-11, a small-molecule caspase inhibitor, has been selected as a candidate for further evaluation and clinical development. It was well tolerated in all subjects accompanied by an average effective dose at 0.025 ± 0.004 mSv/MBq [29]. The elimination of this radioligand was mainly through the kidney and hepatobiliary system, making the gallbladder wall, small intestine, upper large intestinal wall, urinary bladder wall, and liver the five organs receiving the highest dose of activity. Owing to the ability to specifically distinguish apoptotic and necrotic cells, ^18^F-ICMT-11 was considered as an apoptosis developer suitable for human bodies, whose uptake was pH-independent. However, as a caspase-3 inhibitor, ^18^F-ICMT-11 was thought to have limited binding sites, and its accumulation was mainly dependent on active caspase concentration as well as the affinity of the inhibitor to these caspases [9]. This may lead to a low baseline uptake of radioactivity. Nowadays, a number of ^18^F-ICMT-11 analogs, including FITI, have been synthesized and investigated preclinically in order to obtain probes with much more favorable biodistribution properties [30, 31]. Furthermore, the development of radiolabeled caspase substrates was also expected to solve the problem of binding site saturation.

^18^F-CP18 was the first substrate-based apoptosis targeting peptides for PET imaging in clinical trials. After injection, it can be rapidly cleared from the blood pool and excreted via the renal system [32]. Therefore, the highest ^18^F-CP18 activity was discovered in the urinary bladder wall, while frequent urination may cause a reduction of the effective dose (from 38 ± 4 µSv/MBq over a 4.8-h voiding interval to 15 ± 2 µSv/MBq over a 1-h voiding interval). Compared with caspase inhibitors, radiolabeled caspase substrates owned their unique advantages, such as no problem of binding site saturation and, in theory, signal amplification [9]. However, it has been demonstrated that after injection of ^18^F-CP18, the radioactivity concentrations in target tissues were usually very low, probably because the substrate was slowly diffused into tissues and cells [33]. Therefore, further optimization of caspase-targeted developer was required. Currently, ^18^F-C-SNAT4, an improved PET tracer targeting caspase-3, has already been developed and utilized to monitor therapeutic response in mouse models [34–36]. It displayed improved serum stability and high sensitivity for apoptotic cells, which may facilitate its clinical translation. Indeed, a phase I/II study (NCT04017819) has been undergoing to discover the biodistribution and pharmacokinetic of this radiotracer in patients with lung cancer. However, the clinical data has not been reported yet.

### Apoptosis imaging in tumor

A great number of preclinical data supported the viewpoint that apoptosis played an important role in tumor growth, migration, and metastasis. Up to now, apoptosis imaging has been widely applied in clinical oncology trials, for its sufficient intra-observer, inter-observer, and day-to-day reproducibility [37].

#### Apoptosis imaging in tumor: identification of apoptosis in malignant tumors

Uncontrolled proliferation has long been considered as the essence of neoplastic disease. Cancer cells needed large-scale biosynthetic and bioenergetic programs to survival, growth, and division. However, the rate of intra-tumoral neovascularization was far from sufficient to provide the energy required for cancer cells, resulting in a significant rate of apoptosis especially at the central portion of the tumor. Thus, the high apoptotic index discovered in malignant tumors may be helpful in tumor diagnosis [38]. Especially, when it comes to brain tumors, it should be noticed that the permeability of blood–brain barrier (BBB) was usually destroyed by malignant tumors. This may lead to more radiotracer accumulation in baseline scan compared to benign lesion without BBB alteration, which explained the application of apoptosis tracer in the intracranial tumor diagnosis.

**Fig. 5:**
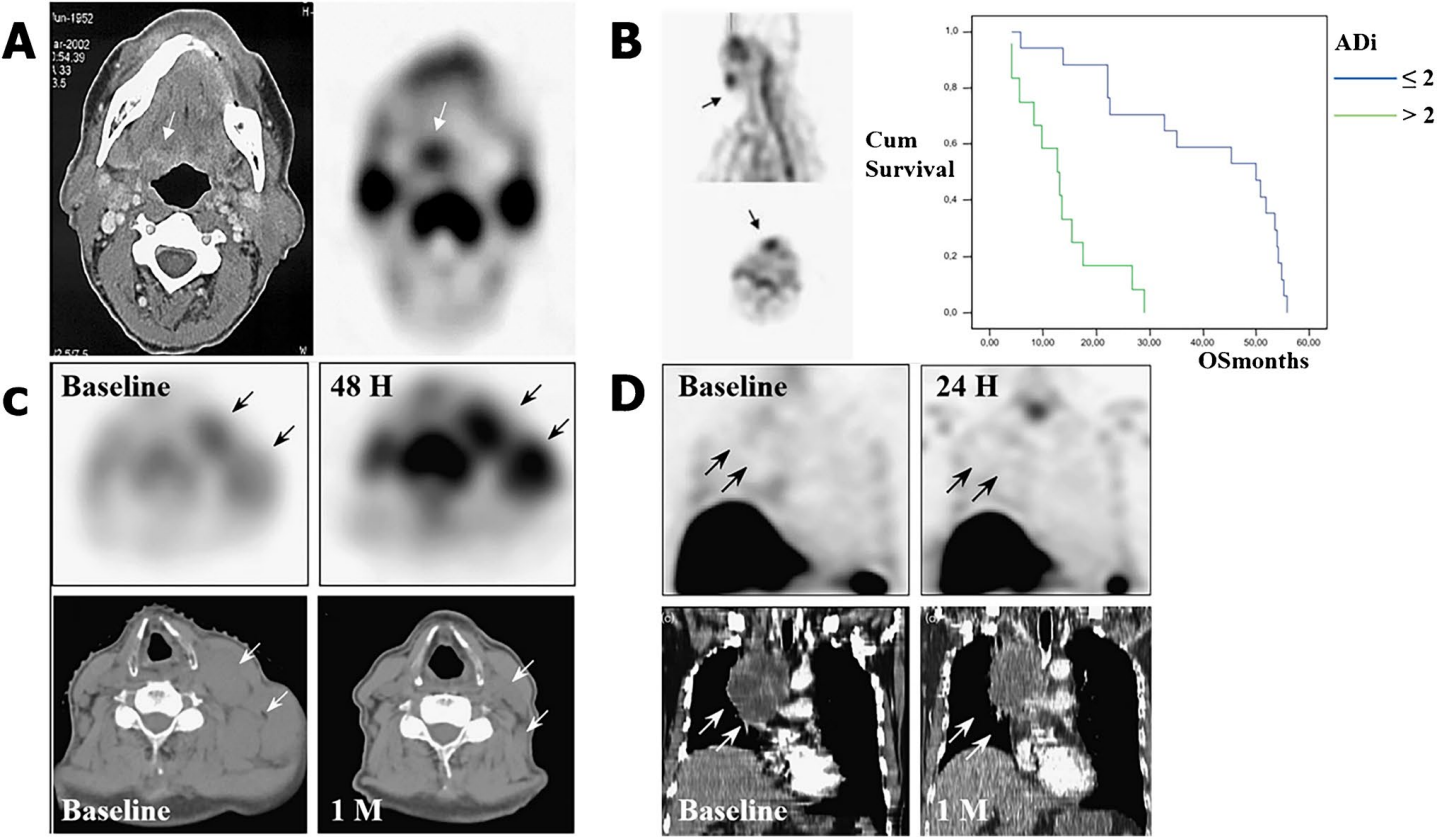
Radionuclide imaging of apoptosis can be served as diagnostic markers, early-response indicators, and prognostic predictors in tumor. **A** A spinocellular carcinoma of the tongue basis in CT and corresponding ^99m^Tc-Annexin V SPECT, indicated by small arrows. **B** A primary tumor located in the lip is showed in ^99m^Tc-Annexin V SPECT (left, indicated by small arrows) along with a Kaplan–Meier plot of overall survival (right). **C** ^99m^Tc-Annexin V SPECT early after treatment shows a significant increase in tumor uptake (black arrows) with a complete response on CT scan 1 month later (white arrows). **D** ^99m^Tc-Annexin V SPECT early after treatment shows a decrease in tumor uptake (black arrows) with a stable disease on CT scan 1 month later (white arrows). (The researches were originally published in Nucl Med Commun. 2004 Mar;25(3):259–63 [39], Eur J Nucl Med Mol Imaging. 2008 Jan;35(1):47–52 [56], Nucl Med Commun. 2008 Jan;29(1):39–44 [48], respectively. Reprint permission was obtained)

^99m^Tc-Annexin V was proficient in quantitative detection of apoptosis in tumor. Hubert Vermeersch et al. [39] demonstrated that in the patients suffering from squamous cell carcinoma of the head and neck (SCCHN), ^99m^Tc-Annexin V activity in tumor lesion (%ID/cm^3^) was correlated linearly with its Fas ligand (FasL) expression (HSCORE) (Fig. 2). Similar correlation was also discovered between ^99m^Tc-Annexin V tumor activity (%ID/cm^3^) and the apoptotic cell numbers calculated by TUNEL assays [40]. Nevertheless, further discussions were needed to verify the potentiality of ^99m^Tc-Annexin V for identifying malignant tumors. In another study, Hubert Vermeersch and colleagues assessed ^99m^Tc-Annexin V scintigraphy findings in 18 patients with head and neck carcinoma and compared them with CT and histology results [41]. They pointed out that although they failed to detect most of the involved lymph node, ^99m^Tc-Annexin V was allowed for a straightforward visualization of all primary head and neck tumors that can be identified by CT scan (Fig. 5A). Except for ^99m^Tc-Annexin V, PET/CT imaging with ^18^F-ML-10 was also found to be capable of visualizing apoptosis in brain cancers and discriminate them from benign lesions. It has been confirmed that baseline ^18^F-ML-10 PET/CT showed an elevated radioactivity in the GBM site, with the highest tracer uptake at tumor center [42].

#### Apoptosis imaging in tumor: early assessment of response to therapy

As demonstrated by various researches, tumor cells tended to evade apoptosis for promoting drug resistance and tumor progression. Compared to the baseline values, significant increased accumulation of apoptosis probe at tumor site early after treatment may predict a complete or partial tumor response [43–46]. No remarked post-therapy tumor uptake, on the contrary, may suggest a futile treatment or even a progressive disease (Fig. 5C and D). Therefore, apoptosis imaging was also widely used to monitor the effect of antitumor treatment, which was helpful in selecting the best therapeutic approach [38].

^99m^Tc-Annexin V imaging has been widely applied in oncology trials for evaluating the effectiveness of multiple treatments in recent years. Sylvie Rottey and colleagues calculated the change value of tumor-to-background activity ratio pre-/post-treatment using sequential ^99m^Tc-Annexin V imaging protocol. Via a 25% change threshold, they successfully separated chemotherapeutic treatment responders from nonresponders in an accuracy of 94% [47]. In another study, ^99m^Tc-Annexin V scintigraphy was utilized to assess the radiotherapy efficacy in follicular lymphoma (FL) patients, and the optimal time window of ^99m^Tc-Annexin V imaging was determined by sequential fine-needle aspiration cytology [44]. It has been verified by Haas et al. that it would be most appropriate to visualize therapy-induced apoptosis within 24–48 h after the last radiation treatment. Moreover, when scored in a semiquantitative manner, increased tumor uptake after radiotherapy showed a favorable correlation with the clinical outcome. Interestingly, using a four-step scale of visual evaluation, the association between the changes of ^99m^Tc-Annexin V tumor uptake (ATU) and therapy outcome can also be discovered [48]. These results altogether indicated that apoptosis imaging with ^99m^Tc-Annexin V was a reliable and reproducible method for efficacy assessment. Notably, its ability to identify treatment-induced apoptosis in normal structures was also reported, which may help evaluate dose–effect relations in various treatment schedules [49].

Compared to SPECT, PET had a better sensitivity and enabled more accurate quantitative evaluation of tracer assimilation [8]. Being the first PET radiotracer for apoptosis imaging which has entered the clinical stage, ^18^F-ML-10 exhibited promising results in some small-scale clinical practices. In a patient suffering from glioblastoma multiforme (GBM), the uptake of ^18^F-ML-10 around the tumor periphery was observed to have an increasement early after therapy, suggestive for treatment-triggered tumor cell apoptosis [42]. Similar conclusion was also found in a phase II study, in which a highly significant correlation between early changes on tumor ^18^F-ML-10 uptake and subsequent tumor anatomical dimensions alterations was discovered through voxel-based analysis of ^18^F-ML-10 PET images [50]. In fact, it has been confirmed that heterogeneous changes of apoptosis in intracranial tumors can be observed as early as 48 h after CyberKnife (CK) therapy. The regression equation of the alteration in radioactivity (*X*) and subsequent tumor volume (*Y*) was *Y* = 1.018 × *X*-0.016 (*r* = 0.862, *p* < 0.05), emphasizing the great potentiality of ^18^F-ML-10 in early prediction of tumor anatomic response (Fig. 3).

^18^F-ICMT-11 was another PET probe now under clinical translation. Dubash et al. wondered whether it was capable of measuring chemotherapy-induced caspase 3/7 activation in breast and lung cancers [51]. In patients with lung cancer, it has been depicted that without the presence of significant perfusion alterations, ^18^F-ICMT-11 PET changes were concordant with cell death. This strongly highlighted the potential use of ^18^F-ICMT-11 PET as a promising candidate for noninvasive imaging of caspase-3/7 activation. However, in breast tumors, the SUVmax of ^18^F-ICMT-11 at baseline was low and remained unchanged following treatment, probably due to the low caspase-3 levels which tremendously limited the sensitivity of ^18^F-ICMT-11 PET imaging.

Moreover, using in vitro and in vivo preclinical models, ^18^F-CP18 has been testified to visualize tumor cell apoptosis triggered by treatment and reliably predict therapeutic efficacy [52, 53]. Notably, in addition to tumor cells, chemotherapeutic also induced apoptosis in normal structures, thus resulting in ineluctable side effects, such as cardiotoxicity and myocardial dysfunction caused by anthracycline. ^18^F-CP18, useful for detection of myocardial apoptosis, may be served as a valuable tool for cardiac risk stratification in patients undergoing anthracycline-based chemotherapy [54]. Next, well-designed clinical trials were expected to verify these conjectures in humans.

#### Apoptosis imaging in tumor: prediction of overall or progression-free survival

Tumor response to therapy has long been demonstrated to closely correlate with the clinical outcome. The visualization of apoptosis may contribute to the identification of effective therapies and the acquisition of valuable prognostic information [38]. ^99m^Tc-Annexin V was injected into patients with lung cancer, lymphoma, or breast cancer for imaging chemotherapy-induced apoptosis immediately after the first course of chemotherapy [55]. The results demonstrated a remarkable relevance between the tumor uptake of ^99m^Tc-Annexin V 24–28 h after treatment and the overall/progression-free survival in patients suffering from lung cancers and lymphomas. Moreover, a high apoptotic index of malignant tumors in baseline scan was also considered to imply a poor prognosis (Fig. 5B). It has already been confirmed that pre-treatment tumor-to-background ratios (T/N) of ^99m^Tc-Annexin V activity can provide independent prognostic information on disease-free survival and overall survival in patients suffering from primary head and neck tumors [56].

According to the clinical trials mentioned above, both baseline and sequential ^99m^Tc-Annexin V imaging can classify the risk stratification of tumor patients as well as assess their overall outcome. The difference was that baseline scan was served as the independent prognostic factor for survival, whereas sequential images mainly speculated survival rates by predicting treatment efficacy. Besides, when baseline ^99m^Tc-Annexin V uptake was not observable in some cases, such as lung cancer, lymphoma, and breast cancer, sequential images may be the only option.

### Apoptosis imaging in cardiovascular disease

Apoptosis was also known to participate in the classical pathological processes of cardiovascular disease. Using radionuclide imaging of apoptosis, abundant preclinical trials have been performed in the assessment of atherosclerotic plaques [57, 58], aortic aneurysm (AA) [59], ischemia–reperfusion injury [60], and their therapy effectiveness [61–64]. Some of them have been conducted in humans and obtained positive results.

**Fig. 6:**
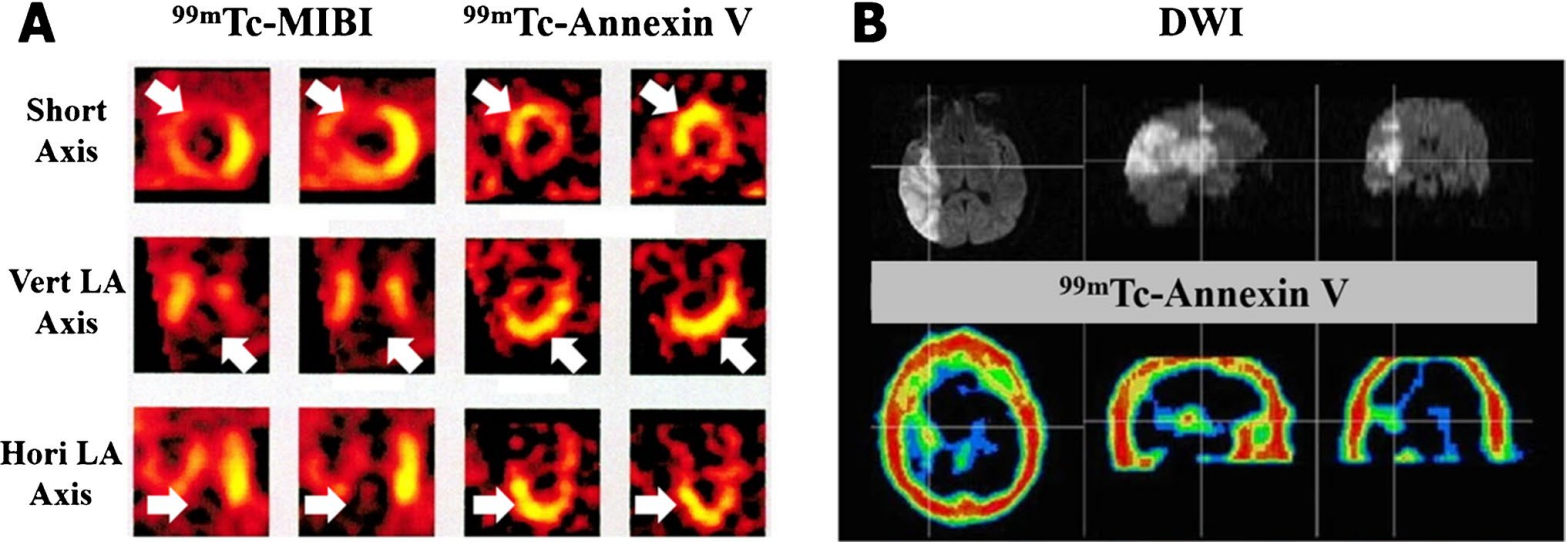
**A** ^99m^Tc-MIBI and ^99m^Tc-Annexin V SPECT of a patient with AMI. **B** DW1 and ^99m^Tc-Annexin V SPECT of a patient with acute stroke. (The researches were originally published in J Nucl Med. 2003 Mar;44(3):391–6 [65] and Eur J Nucl Med Mol Imaging. 2006 May;33(5):566–74 [69], respectively. Reprint permission was obtained)

Previous clinical studies have revealed that ^99m^Tc-Annexin V SPECT/CT played an important role in the diagnosis and assessment of acute myocardial infarction (AMI) [65, 66], heart failure [67], cardiac allograft rejection [68], and so on. Thimister et al. used ^99m^Tc-Annexin V scintigraphy to determine the risky areas in patients with AMI and compared ^99m^Tc-Annexin V images with those using ^99m^Tc-MIBI (Fig. 6A). As a result, all patients showed remarkable ^99m^Tc-Annexin V accumulations at the infarct site in concordance with the perfusion defects on ^99m^Tc-MIBI scintigraphy [65]. When injected into patients suffering from acute stroke (Fig. 6B), ^99m^Tc-Annexin V was confirmed to depict the in vivo region of ischemic neuronal injury and matched well with sites of restricted diffusion on MRI [69, 70]. Notably, healthy volunteers were also recruited and underwent preconditioning for assessing therapeutic effects of ischemia and reperfusion injury using ^99m^Tc-Annexin V [71–77]. When it comes to atherosclerosis, it has been reported that plaque rupture was closely linked to the macrophages and smooth muscle cells apoptosis in the plaque. Therefore, molecular imaging with ^99m^Tc-Annexin V may be a promising way to evaluate plaque instability and discern patients who had a risk of acute vascular events [78]. Similar prognostic value was also reported in arrhythmogenic right ventricular cardiomyopathy/dysplasia (ARVC/D) in which apoptosis has been proposed to mediate persistent loss of heart muscle cells and then ventricular dysfunction [79].

Interestingly, scientists also considered that due to its small molecular size and minimal number of functional groups, ^18^F-ML-10 may own the ability to cross the intact blood–brain barrier and enable the imaging of neurons loss after stroke [80, 81]. Moreover, since cardiomyocyte apoptosis was involved in the cardiac remodeling processes and heart failure progression in myocardial infarction, it was not surprising that imaging apoptosis by ^18^F-ML-10 PET/CT in patients suffering from cardiovascular disease may help detect the disease progression and provide treatment guidance [28]. However, all these functions were waiting to be verified in human bodies.

### Apoptosis imaging in neurodegenerative diseases

One of the characteristics of neurodegenerative diseases was the aberrant activation of PCD pathway, including apoptosis, resulting in unwanted loss of neuronal cells [82]. Thus, radionuclide imaging of apoptosis can be applied in neuroimaging research to noninvasively visualize the apoptosis of neuron in the brain, enabling identification and real-time assessment of neurodegenerative diseases.

Examining PS expression within the cerebrum of AD patients, Y. Lampl and colleagues identified a multifocal cortical uptake of ^99m^Tc-Annexin V in these patients and conjectured that it may provide complementary informations for differentiating AD from non-AD dementia [83]. In addition, using cellular models of Parkinson’s disease (PD), the feasibility of ^99m^Tc-Annexin V for detecting early neuron damage in PD was confirmed, although lacking clinical validation [84].

Notably, ^18^F-ML-10 was also considered as a suitable probe for clinical application in neurodegeneration diseases [9] as it can cross the BBB to image the loss of neurons. However, this notion has not been verified in human bodies.

### Apoptosis imaging in other diseases

Clinical studies with apoptosis imaging have also been implemented for the detection of cell death in other diseases. As early as 2009, Lorberboym et al. have described the differential diagnostic role of ^99m^Tc-Annexin V SPECT/CT in distinguishing prosthetic infection with aseptic loosening [85]. As for Crohn’s disease, since antitumor necrosis factor (TNF) antibody infliximab functioned as an inducer of activated lamina propria T lymphocyte apoptosis, it was not surprising that imaging apoptosis by ^99m^Tc-Annexin V SPECT/CT in patients suffering from inflammatory bowel disease may help predict the efficacy of anti-TNF therapy [46].

The monitoring role of ^18^F-ML-10 PET/CT in assessing the fibrotic activity of pulmonary fibrosis was also demonstrated [86, 87]. Additionally, when it has been corroborated that proapoptosis drugs can also be utilized as host-directed therapies in tuberculosis (TB) patients, ^18^F-ICMT-11 may be a noninvasive approach for in situ measurement of intralesional therapeutic response in TB [88]. Unfortunately, both required clinical validation.

## Conclusion and prospection

In the past decades, scientists have made great efforts in developing radiolabeled apoptosis targeting agents, which was allowed for repetitive and selective in vivo identification of apoptotic cell death without the need of invasive biopsy. To date, some of radiopharmaceuticals, such as ^99m^Tc-Annexin V, ^18^F-ML-10, ^18^F-CP18, and ^18^F-ICMT-11, have been synthesized and under clinical practices on multiple diseases. Since aberrant cell death (either resisting or excessive) was served as a natural hallmark to the development of some diseases, such as tumor, ischemic injury, and neurodegenerative diseases, noninvasive monitoring of apoptosis through these tracers may provide important clinical information that can help tailor treatment. Firstly, it has been depicted that apoptosis imaging markers may have diagnostic value for their ability to identify the course and progression of diseases. Besides, the potentiality of apoptosis imaging as an early indicator of the treatment response was also confirmed. Finally, apoptosis imaging may also be able to classify the risk stratification of patients as well as evaluate their overall outcome independent of treatment.

However, almost all the existing radiolabeled probes posed their defects. ^99m^Tc-Annexin V, for instance, was criticized for its nonspecific uptake as well as inability to distinguish between apoptosis and necrosis [51]. On the contrary, although capable of discriminating apoptosis from other types of cell death, how ^18^F-ML-10 specifically targeted apoptotic cells remained undefined, which inevitably challenged its clinical practice [9]. As for radiotracer targeting activated caspases, ^18^F-ICMT-11 was limited by binding site saturation and ^18^F-CP18 by a low radioactivity in target tissues [9]. Besides, except for ^99m^Tc-Annexin V, other radioligands were lack of clinical application in non-tumor fields. Thus, improved radio-probes and well-designed prospective trials were required to validate the great clinical value of apoptosis imaging as diagnostic markers, early-response indicators, and prognostic predictors. Only in this way, one or more of these imaging agents may eventually develop to be authorized and widely utilized in clinic.
